# Mechanisms of Autoimmune Cell in DA Neuron Apoptosis of Parkinson's Disease: Recent Advancement

**DOI:** 10.1155/2022/7965433

**Published:** 2022-12-14

**Authors:** Zijian Zheng, Shushan Zhang, Hanwen Zhang, Zhongzheng Gao, Xiangrong Wang, Xinjie Liu, Cheng Xue, Longping Yao, Guohui Lu

**Affiliations:** ^1^Department of Neurosurgery, The First Affiliated Hospital of Nanchang University, Nanchang, China; ^2^Department of Neuroanatomy, Group for Regeneration and Reprogramming, Institute for Anatomy and Cell Biology, Medical Faculty, Heidelberg University, 69120 Heidelberg, Germany

## Abstract

Parkinson's disease (PD) is a prevalent neurodegenerative disorder that manifests as motor and nonmotor symptoms due to the selective loss of midbrain DArgic (DA) neurons. More and more studies have shown that pathological reactions initiated by autoimmune cells play an essential role in the progression of PD. Autoimmune cells exist in the brain parenchyma, cerebrospinal fluid, and meninges; they are considered inducers of neuroinflammation and regulate the immune in the human brain in PD. For example, T cells can recognize *α*-synuclein presented by antigen-presenting cells to promote neuroinflammation. In addition, B cells will accelerate the apoptosis of DA neurons in the case of PD-related gene mutations. Activation of microglia and damage of DA neurons even form the self-degeneration cycle to deteriorate PD. Numerous autoimmune cells have been considered regulators of apoptosis, *α*-synuclein misfolding and aggregation, mitochondrial dysfunction, autophagy, and neuroinflammation of DA neurons in PD. The evidence is mounting that autoimmune cells promote DA neuron apoptosis. In this review, we discuss the current knowledge regarding the regulation and function of B cell, T cell, and microglia as well as NK cell in PD pathogenesis, focusing on DA neuron apoptosis to understand the disease better and propose potential target identification for the treatment in the early stages of PD. However, there are still some limitations in our work, for example, the specific mechanism of PD progression caused by autoimmune cells in mitochondrial dysfunction, ferroptosis, and autophagy has not been clarified in detail, which needs to be summarized in further work.

## 1. Introduction

Parkinson's disease (PD) is a central neurodegenerative disease second only to Alzheimer's disease. The incidence of PD ranges from 10 to 18 per 100000 person-years [1, 2]. The main pathological manifestations of the disease were DArgic (DA) neuron apoptosis and deposition of *α*-synuclein protein in the Substantia Nigra (SN). When the apoptosis of DA neurons accounts for more than 50% of the total DA neurons, patients can develop clinically assessable motor symptoms. [3]. A complete understanding of the mechanism of DA neuron apoptosis may be the key to the research of PD therapy.

In the past, the role of the autoimmune system in PD has not been valued [4]. However, data accumulated over the past decade regarding immune alterations in PD increased the interest to pursuing such an association [5]. Immune-related genes and antigen molecules play a particular role in DA neuron apoptosis. Herein, we present a comprehensive review of the impacts of autoimmunity in PD. We have composed a logical argument to substantiate that autoimmunity is actively involved in the pathogenesis of DA apoptosis in PD through several proteins, including *α*-synuclein, DJ-1, PINK1, and parkin [6–8], as well as autoimmune cells, such as B cells and plasma cells, conventional CD4+ and CD8+ T cells, microglial cell, dendritic cell, and NK cell [9, 10]. Furthermore, based on the relationship between autoimmune cells and their related genes and antigen molecules on the DA neuron apoptosis in PD, we put forward our own opinions for the research and clinical treatment of the disease.

Despite the potential significance of autoimmune cells in the development of PD, there is a lack of discussion about them. Therefore, the aim of this study is to conduct a systematic review of autoimmune cells in PD to further explore them. In this review, we consider the link between autoimmune cells and PD and summarize the role of autoimmune cells in PD and discuss their role in DA neuron apoptosis based on the extensive literature. We hold the opinion that alterations in autoimmune cells may contribute to one of the key mechanisms in PD.

## 2. Immune Landscape in PD

More and more studies have shown that PD is an autoimmune disease, when the immune mechanism is out of balance, the autoimmune system mistakenly attacks normal cells, including DA neurons. For PD, chronic autoimmune attacks play an important role in the early stage of the disease and participate in the whole development. Autoimmune disorders will first lead to the upregulation of neuroinflammation and then, with the participation of various autoimmune cells and immune-related molecules, induce the apoptosis of DA neurons and accelerate the progression of PD [6]. The involvement of B cells in the above processes has been found more and more widely, while there is not much research on plasma cells.

It is undeniable that many previous studies have described the changes in the number and subsets of B cells in PD patients. Still, few studies reported the correlation between B cells and PD progression [11]. With the progress of research, people increasingly find that there may be a special relationship between this change and the progression of PD. First, in terms of quantity, there was an overall decrease of B cells in PD patients compared with healthy controls, especially in immature B cells [12]. By detecting the number of T cells, B cells, NK cells, and monocytes in peripheral blood, it was found that CD4+ T and CD19+ B cells decreased slightly (15-25%) in patients with PD [13], and the decrease of CD3+, CD4+, CD8+ T, and B cell was associated with the progression of PD [14]. In the study of PD model mice of different ages, it was further observed that CD3+ T cells and CD4+ Th cells increased and CD19+ B cells decreased in 2-month-old A53T mice, which was related to a mood disorder in mice. The number of CD4+ Th cells, CD3+, CD4+, and CD25+ T regulatory cells in 10-month-old mice increased, which was related to movement disorder in mice, and B cells had no significant change [15]. In addition, an increase in the number of *α*-synuclein-specific B cells was found in the peripheral blood of PD [16], suggesting that B cells may also be involved in the pathological process of *α*-synuclein-related PD. The above arguments prove that there are changes in the number of B cells in patients with PD and are related to the overall progress of PD.

In the study of the changes of B cell subsets in PD, some scholars reported that the number of proliferative B cells in PD patients was lower than that in normal controls. The proportion of B cell subsets with regulatory function decreased, such as transitional B cells. In contrast, the production of B cells producing inflammatory factors increased, enhancing proinflammatory function of B cell. In addition, the principal component analysis also showed that the expression of TNF-*α* and GM-CSF in B cells and T cells of PD patients was increased. In addition, the decrease of follicular T cells, an essential group of B cell helper T cells, has also been proven to be related to B cell abnormalities. The study also shows that B cells are the first to be affected in the progression of PD [17]. It has been proved that the numbers and subset changes in B cell may participate in the pathological process of PD and profoundly affect the progression of the disease.

Autoimmune cells are found in the brain parenchyma, cerebral spinal fluid, and meninges, which can be regarded as either induction factors or protective factors of neuroinflammation [18, 19]. Studies have shown that peripheral antigen-presenting cells (APCs) could migrate across blood-brain barrier (BBB) and intake *α*-synuclein accumulates in the substantia nucleus (SN) of PD [20], and then present them to CD4+ and CD8+ T cells, so *α*-synuclein serve as a major antigen to active antigen-specific T cells [20]. Once T cells recognize the antigens, the local adaptive immune response can be induced. T cells will differentiate into a variety of effective T cells, including Th1, Th2, Th17, follicular helper T cells (Tfh), and Tregs. Among these effector subsets, Th1, Th2, and Th17 cells drive proinflammatory responses while Tregs exert anti-inflammatory and immunosuppressive activities. Th1 secretes IL-2, interferon-gamma (IFN-*γ*), and tumour necrosis factor (TNF); Th2 secretes IL-4, IL-5, IL-10, and IL-13; Th17 secretes IL-17, and Tregs secrete IL-10 and transforming growth factor-beta (TGF-*β*) [21]. All these immune factors will consequently reversely recruit APCs, so it leads to a malignant circulation of the severe immune response.

A balance between pro- and anti-inflammatory immune responses is essential to maintain generalized homeostasis, especially within the CNS, where an imbalance immune responses can lead to disease [22]. The CD4+ T and CD4/CD8 ratio in cells is significantly decreased in PD patients compared with healthy controls, indicating an immune disorder in PD. However, the T cell clonally increased in CSF of PD patients. Among the CD4+ T cells, a group of cytotoxic CD4+ T cells (CD4 CTLs) dramatically clonally increased in PD patients, and these CD4 CTLs were validated to be differentiated from Th1 cells [23], Li et al. also found that PD patients had more Th1 cells compared with healthy volunteers [24]. Additionally, cell frequencies and absolute numbers of naive CD4 T cells, gamma delta T cells (*γδ*T), and iNKT cells were significantly decreased in groups with PD [25, 26]. Another subset of CD4+ T cells, cTfh and cTfr cells, may be connected with the chronic progression of PD; cTfh is crucial for proinflammation through promoting the differentiation of B cells into high-affinity plasma cells and the formation of germinal centers, while cTfr plays a negative role in both B cell activation and neuroinflammation. The proportion of cTfh cells among CD4(+) T cells in PD patients was significantly higher than that in HCs, while cTfr cells in PD patients were slight decreased [27].

Moreover, patients with PD had a higher proportion of Treg cells both in periphery blood and SN [28]. Increased Treg cells might indicate the effort of the immune system to suppress ongoing neuroinflammation [29]. Autoimmune cells also vary along with the progression of PD; longitudinal research validates the quantity of CD8+ T cells that were various in different stages of PD. In the earliest stage of the disease, when SN *α*-synuclein aggregation is absent, a robust CD8+ T cell infiltration and no DA neuron apoptosis were found; in the next stage, CD8+ T cell infiltration is milder while *α*-synuclein accumulated. Subsequently, their density of CD8+ T cells positively correlated with neuronal death [29]. In conclusion, it indicates that more proinflammatory T cells may across the BBB into the brain parenchyma and contribute to DA neuron apoptosis suffering more intensive immune response; however, the specific mechanism between T cells and DA neuron apoptosis needs further research (Table 1).

In 2012, Anderson et al. reported that the killer cell immunoglobulin-like receptor (KIR) in NK cells decreased significantly with the aggravation of stiffness and gait disorder in PD patients [30]. A study compared the number of NK cells in the blood of PD patients with non-PD patients and found that the number of NK cells in the blood of PD patients was significantly higher than that of the control group [31]. Recently, it has been reported that in PD's preclinical mouse model, the depletion of NK cells results in an increase in *α*-synuclein in many brain regions, including the striatum, SNpc, and brainstem. This suggests that NK cells may be associated with the pathogenesis and progression of PD, and this association may be protective. It was found that NK cells were close to *α*-synuclein aggregates using an immunohistochemical technique to analyze the brain tissue after the death of PD and PD dementia [32].

## 3. Mechanism of DA Neuron Apoptosis

The DA neurons in SN play an essential role in maintaining the human brain's normal sensation, movement, emotion, and cognition. The abnormality of its structure and function is closely related to various neurodegenerative diseases. Up to now, studies have shown that DA neuron apoptosis is the main factor in the pathological progress of PD, and its mechanisms mainly include *α*-synuclein misfolding and aggregation, apoptosis, autophagy, mitochondrial dysfunction, oxidative stress, and neuroinflammation.

Studies have shown that *α*-synuclein plays a vital role in DA neuron apoptosis, and its structure and function have been widely studied [33]. One of the main pathological features of PD is the widespread protein inclusion bodies in SN neurons, namely, Lewy bodies (LBs) and Lewy neurites (LNs) [34], which are mainly composed of filamentous aggregates containing phosphorylated and ubiquitin *α*-synuclein proteins. Among them, the most direct evidence shows that the aggregation of *α*-synuclein under pathological conditions is the most direct toxic medium of DA neuron apoptosis, which can directly cause DA neuron apoptosis and promote the onset and development of PD [35, 36]. Secondly, apoptosis, mainly characterized by cell size reduction, cytoskeleton collapse, and nuclear pyknosis, is also widely studied in PD [37–39]. For example, the caspases pathway has been widely and deeply involved in apoptosis-mediated DA neuron apoptosis response [40, 41]. Subsequently, autophagy was found abnormal in DA neurons as the main pathway of intracellular protein degradation and maintenance of cell homeostasis. Ultrastructural examination of autophagosomes in the brain of patients with PD revealed a large number of phosphorylated erk-labelled mitochondria, suggesting that DA neuron apoptosis was related to abnormal mitochondrial autophagy [42].

In recent years, more and more new mechanisms have been found to play a role in DA neuron apoptosis, and mitochondrial dysfunction is considered the core of the pathogenesis of sporadic and familial PD. Knockout of PINK1 in DA neurons of human and PD model mice can lead to a wide range of mitochondrial dysfunction, including abnormal mitochondrial morphology, a decrease in membrane potential and an increase in ROS production, resulting in DA neurons susceptible to apoptosis [43]. More and more research results show that microglia-mediated neuroinflammation seems to be one of the most critical mechanisms of DA neuron injury; studies have found that microglia activation and NF-*κ*B nuclear translocation can be induced by pathological factors and further promote the release of inflammatory cytokines under the action of autophagy and other mechanisms [44, 45], resulting in neuroinflammation and DA neuron apoptosis [45].

Chronic oxidative stress is a critical factor in DA neuron apoptosis [46]; it can induce the accumulation of *α*-synuclein and lead to the impairment of DA neurons. For instance, it has been reported that under oxidative stress, SNCA mutations in PD patients lead to a significant accumulation of *α*-synuclein in Lewy bodies and accelerate disease progression [47, 48]. At the same time, misfolding and aggregation of *α*-synuclein can, in turn, increase the production of ROS and aggravate the process of oxidative stress [49, 50]. First, in CD8+T cells, MPP+/MPTP can accelerate the oxidative stress, then trigger major histocompatibility complex class I (MHC-I) presentation and CD8+ T cells activation, and finally aggravate the immune damage susceptibility and degeneration of DA neurons. It suggests that oxidative stress can accelerate the destruction of DA neurons by CD8+T cells; this may be one of the mechanisms of which oxidative stress induces PD [51]. In addition, the role of activated microglia mediated DA neuron apoptosis is also attributed to oxidative stress. Thus, *α*-synuclein-accumulated microglial cells developed a strong reactive state with excessive production of oxidative and proinflammatory molecules. This inflammatory state created more DA neuron apoptosis. Pharmacological inhibition of oxidative and nitrosative molecule production was sufficient to attenuate neurodegeneration [52]. These results suggest that oxidative stress in microglia induces DA neuron apoptosis by promoting an excessively inflammatory environment and the selective recruitment of autoimmune cells. However, we found no direct evidence for this in B cell. More studies will be needed to further explore the link between oxidative stress and B cell induces DA neuron apoptosis.

## 4. Autoimmune Cells in the Pathogenesis of DA Neuron Apoptosis in PD

### 4.1. B Cells

There are different opinions on whether B cell is an essential regulator of DA neuronal apoptosis. In a study on the regulation of autoimmune cells in A53T-*α*-synuclein PD model mice, it was observed that T cells could cause DA nerve degeneration and promote PD. Still, no degeneration of DA neurons was observed in the downregulated B cell model mice [53]. Some studies have also found that 6-OHDA injection can significantly increase B cells in the SN of rats at the injection side, which may be related to extensive DA neuronal destruction [54]. In recent years, the relationship between B cell and PD progression and DA neuronal apoptosis has gradually deepened, and a breakthrough has been made in PD-related genes and autoimmune antibodies.

In addition, researchers also found that increasing the level of DA in the body can promote the differentiation of B cells into plasma cells [55], suggesting that plasma cells may be related to the DA system, but we have not found more evidence for it.

#### 4.1.1. Gene-Mediated Apoptosis Related to B Cells

*(1) LRRK2*. The general decrease of B cell level in PD patients may be associated with the change in B cell-related gene expression. LRRK2 (leucine-rich repetitive serine/threonine protein kinase 2) is reported to be overexpressed in B cells of PD and may be associated with B cell functional changes [45, 56, 57].

It was found that lipopolysaccharide (LPS) could induce the increase of LRRK2 gene expression and protein kinase activity in cells with a time-dependent manner. Specifically, LPS can activate the Toll-like receptor signal pathway, increase the TRAF6/LRRK2 and phosphorylation level of MAPK (JNK1/2, p38 and ERK1/2), and then promote the release of inflammatory cytokines, which may interfere with the toxic effect of central nervous inflammation on DA neurons. On the other hand, LRRK2 inhibitors can reduce LPS-induced TRAF6/LRRK2 interaction and phosphorylation of MAPK and IkB-*α*, thus reducing the release of inflammatory factors such as TNF-*α* [56]. As B cells can actively express LRRK2, these processes may play an essential role in B cell-mediated DA neuronal apoptosis. As expected, follow-up studies found that LRRK2 mutations in B cells aggravated LPS-mediated neuroinflammation and accelerated DA neuronal loss in the SN [58]. In addition, LRRK2 and CD38 were found to exist in the plasma membrane complex in B cells and act as upstream regulatory molecules of host defence transcription factor TFEB (transcription factor EB) to affect autophagy, which plays a potential role in promoting DA neuron apoptosis [59].

*(2) Parkin*. Parkin mutation is the leading genetic factor in the progression of PD. In B cells, parkin mutation does not directly lead to cell death, but it has been proved to indirectly affect the activity of the DA neural pathway [60]. Studies have shown that the mutation in parkin can increase B cells' sensitivity to DA, iron, and hydrogen peroxide and may promote the process of apoptosis [61]. In addition, manganese ions have also been shown to accelerate the process of apoptosis mediated by parkin mutation; manganese ions can accelerate the cytotoxicity by affecting the cell cycle and promoting DNA apoptosis [62], which may involve the inhibition of mitochondrial function, ATP activity, and downregulation of the caspase3 pathway [63]. In SN, excessive manganese ions can lead to DA neuronal apoptosis and DA pathway disturbance [63, 64]. Therefore, in the pathological process of PD, manganese ions may affect the functional status of parkin-mutated B cells and affect the apoptosis of DA neurons through classical pathways, such as caspase, to achieve synergistic regulation of manganese ions and parkin genes on the progression of PD.

**Figure 1:**
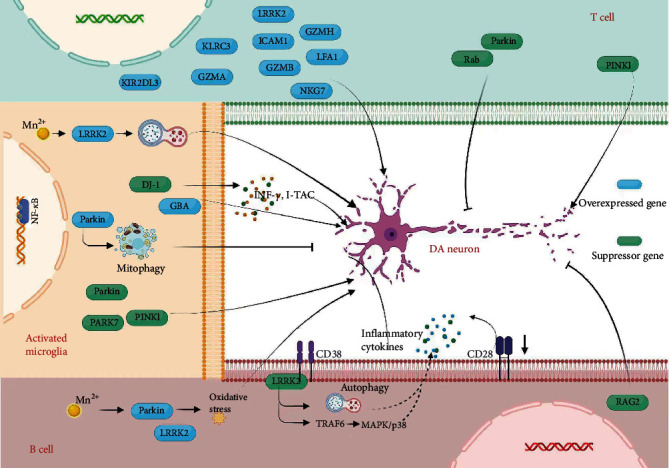
Gene-mediated apoptosis of autoimmune cells in PD. The overexpression (blue) or downregulation of genes (green) in T cells, B cells, and activated microglia is associated with the imbalance of intracellular homeostasis and promotion of inflammation and ultimately leads to DA neuron apoptosis.

Finally, MPTP treatment reduced DA neuron loss and behavioral disorder caused by B cell deficiency in nuclear gene recombination-activated gene 2 (RAG2) knockout mice [65, 66]. In the future, more and more genes may be found to be involved in B cell-mediated DA neuronal apoptosis (Figure 1 and Table 2).

#### 4.1.2. Receptor-Mediated Apoptosis Related to B Cells

Previously, we mentioned that memory B cells in patients with PD increased significantly compared with healthy controls, accompanied by increased expression of MHCII genes and transcription factor activating protein 1 (AP-1), suggesting that antigen presentation ability of B cells in patients with PD is enhanced, which makes it possible for multiple antigens and protein molecules to participate in the progression of B cell-mediated PD [12]. It was found that the expression of lgG and lgA increased in PD patients [72], lgG deposition was also infiltration in DA neurons, and IgG also wrapped the specific Louis corpuscles of PD patients. Still, the relationship between B cell, lgG deposition, and DA neuronal apoptosis in PD patients needs to be further explored [12, 16].

**Figure 2:**
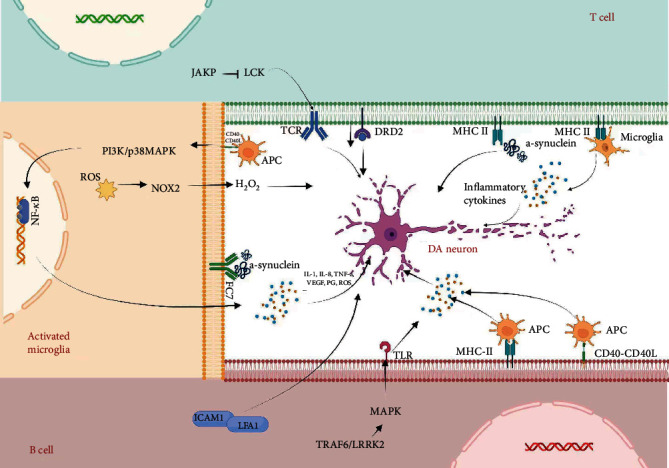
Receptor-mediated apoptosis of autoimmune cells in PD. The different receptors on the autoimmune cell surface recognize various antigens (e.g. *α*-syn) and consequently activate the intensive immune response, including releasing immune factors, which result in DA neuron apoptosis.

Additionally, B cells can upregulate the expression of critical proinflammatory factors through CD40-CD40L interaction and promote the activation of many downstream signal transduction processes, including the recruitment of tumour necrosis factor receptor-related factors (TRAFs), which in turn initiate intracellular signal cascades such as phosphatidylinositol 3-kinase (PI3K), p38MAPK (P38MAPK), NF-kappa B, JunN terminal kinase (JNK), RAS, and Src family kinase pathway [77–79], resulting in the production and release of proinflammatory cytokines, angiogenic factors, prostaglandins, cell adhesion molecules, and chemokines such as IL-1, TNF-*α*, IL-8, vascular endothelial growth factor, ICAM-1, and vascular cell adhesion molecule-1 [80, 81]. It may accelerate the inflammatory reaction of the central nervous system and apoptosis of DA neurons (Table 3) (Figure 2).

### 4.2. T Cells

The involvement of T cells in PD pathogenesis has been found more and more widely. It has indicated the infiltration of CD4+ and CD8+ T cells in the SN of postmortem brains of PD patients. Additionally, in the MPTP mouse PD model, T lymphocyte infiltration was found in the brain parenchyma, where T cells were associated with DA neuron apoptosis [65, 95–98]. Several cell cytotoxic genes, such as GZMA, GZMB, GZMH, and NKG7, were found overexpressed in PD patients' CD4+ T cells [23]. This T cell response to neurodegenerative changes can trigger harmful events, including cytokine receptor-mediated apoptosis, oxidative stress, and autophagy, consequently leading to DA neuron apoptosis and disease development. Recently, studies on the relationship between T cell and PD progression along with DA neuronal apoptosis have been gradually deepened, and breakthroughs have been made in receptor-mediated apoptosis, *α*-synuclein accumulation and replicative senescence.

#### 4.2.1. Receptor-Mediated Apoptosis Related to T Cells

The loss of DJ-1 is a rare cause of the development of early-onset PD [82]. DJ-1 loss of function sensitizes microglia cells to release interferon-*γ* (IFN-*γ*) and interferon-inducible T cell alpha chemoattractant (I-TAC) and causes inflammatory death to DA neurons. Moreover, DJ-1 depletion suggested a critical role in inhibiting immunogen, including a sign of almost doubled nonsenescent T cells. DJ-1 compared with HCs, the potential mechanism may be in connection with decreased oxidative phosphorylation (OXPHOS) and impaired TCR sensitivity in naive CD8+ T cells at a young age, resulting in a reduced aging process in T cell compartments [82]. Additionally, JNK pathway-associated phosphatase (JKAP) activates T cell receptor (TCR) signalling by directly inactivating Lck [99], which has also been reported to be downregulated in PD patients compared to healthy controls and regulate immune/proinflammatory process via promoting Th1 and T17 cell differentiation in PD [67]. Another protective receptor in PD is DA receptor 2 (DRD2). MPTP-induced DA neuron apoptosis was aggravated in CD4+ T cell-specific DRD2-knockout mice, as well as more severe motor deficits and microglial activation; whereas, DRD2 agonist reversed the shift of CD4+ T cells [100]. Additionally, the deletion of DRD3 in CD4+ T cells weakened the differentiation of primitive CD4+T cells into the Th1 phenotype, which accelerated the formation of Th2 cells, while Th17 differentiation was not affected [83]. Moreover, direct protective effects of CTLA-4 and PD-1 on high inflammatory induced DA neurons were demonstrated. The combination of CTLA-4 and PD-1 blocker contributes to T cells reactivation and accelerates the differentiation of microglia into M1 type to mediate the observed neuroinflammation [101]. Genetic deletion of TCRb or CD4 reduces the MHCII response to *α*-synuclein, protecting DA neurons from death in PD [84].

Th17 is a lineage of proinflammatory CD4+ T cells, named after interleukin-17, the main cytokine produced by these cells. The research found that T17 cells induce neuron apoptosis. After coculture with T cells or the addition of IL-17, PD midbrain neurons suffered increased neuronal death due to upregulation of IL-17 receptor (IL-17R), while blocking IL-17R prevented neuronal death [85]. Additionally, the interaction of intercellular adhesion molecule (ICAM1) with its ligand lymphocyte function-associated antigen 1 (LFA1) activated the CD4+/CD8+ T cell's recruitment into the central nervous system resulting in the observed DA apoptosis [68]. Another well-recognized pathway that promotes neuroinflammation is Toll-like receptors (TLRs), including TLR7 and TLR8. Campolo et al. have demonstrated that the downregulation of TLR7 and TLR8 inhibits T cell recruitment in the SN [86].

Similarly, MHC-I exerted a neuronal apoptosis d effect in the MPTP-induced rat models of PD, accompanied by a growth in the infiltration of CD8+ T cells. Its neuron apoptosis d effects were inhibited by silencing the expression of PTEN-induced 1 (PINK1) [51]. In addition to the above confirmed neural injury mediators, many potential receptors for DA neuron apoptosis have been identified. In recent single-cell T cell receptor sequencing studies, some highly expressed genes in each cluster were shown to have significantly higher expression of CD4, and several are cytotoxic genes, such as GZMA, GZMB, GZMH, and NKG7. Similar to CD8+ T cells, KLRC3 and KIR2DL3, which exhibit toxic cell roles in DA, were highly expressed [23]. To conclude, these results provide evidence that T cell receptor-mediated apoptosis could influence DA apoptosis and suggest that specific subsets of patients with a T cell receptor mutation could be more appropriate for immune-targeted therapies (Table 2).

#### 4.2.2. *α*-Synuclein Accumulation Related to T Cells

Over the past several years, many studies have shown that *α*-synuclein is generally distributed in the brains of patients with PD [102]. Researchers have proposed that abnormal misfolding of *α*-synuclein leads to neuroinflammation and lysosomal membrane permeability, contributing to calcium influx and ion homeostasis destruction, resulting in neuronal toxicity and DA neuron apoptosis [103, 104]. Major histocompatibility complex II (MHC-II), an epitope recognized by T cells, has been recently demonstrated to be located in the Y39 and S129 regions of *α*-synuclein [87]. The overexpression of *α*-synuclein results in the upregulation of MHC II [84, 88]. Once T cells combine with the MHC-II of *α*-synuclein, a series of the immune response are activated. Studies have shown that *α*-synuclein promotes the polarization of CD4+ T cells towards Th1 and Th17 phenotype and infiltration of CD4 and CD8 T cells [88], consequently causing the apoptosis of DA neurons in the MPTP-induce cell model [65]. Van der Perren et al. have indicated that LRRK2 ablation inhibits the accumulation of *α*-synuclein due to a decreased microglial activation and CD4 and CD8 T cell recruitment [69]. Above all, it indicates the crucial role of *α*-synuclein in stimulating T cell immune response in DA neuron apoptosis. Reversely, T cell infiltration is necessary for *α*-synuclein-induced neurodegeneration [84, 105].

Autophagy removes many misfolded proteins in cells through a double-membrane crescent-shaped structure of autophagosomes. If autophagy is impaired, misfolded proteins and dysfunctional mitochondria persist and accumulate in the cytoplasm. Thus, *α*-synuclein accumulation probably results from autophagy impairment [69], further contributing to DC antigen presentation to T cells or promoting neuroinflammation and DA neuron apoptosis by damaging the lysosome [106]. Missense mutation of LRRK2 is the predominant cause of PD [107], LRRK2 kinase activity contributes to neuroinflammation via phosphorylating p53 in PD, and the phosphorylation of p53 induces the expression of TNF-*α* [108]. LRRK2 mutation is also associated with autophagy via promoting dendric cells' antigen presentation to CD4+ T cells [109]. Additionally, Ras-related protein in brain (Rab) proteins are crucial in mediating autophagy and lysosomal degradation. Different subtypes of Rab participate in different periods of the endolysosomal pathway by connecting with Beclin1 and LC3, which is associated with the transfer of *α*-synuclein for antigen presentation by DCs [70] (Table 3).

#### 4.2.3. Replicative Senescence Related to T Cells

It is well acknowledged that aging is a significant risk factor for PD [110]. Aging-related immune senescence is probably has relation to the pathogenesis of PD, for which aging is a risk factor [111]. It is dominantly mediated by CD8+ cells. The most apparent phenotype change is the loss of CD 28 and the overexpression of CD 57, which promotes the secretion of proinflammatory cytokines and limits the proliferative ability of autoimmune cells [112–114]. Effector memory T cells reexpressing CD45RA (TEMRA) cells, a sign of age-associated immune dysregulation, were found at low level in the CD4+ and the CD8+ T cell [115]. Meanwhile, the percentage of effector memory T cells reexpressing CD45RA, CD57+CD56− T cells, and CD57+CD56+ T cells was significantly decreased in PD patients [116]. Taken together, immune replicative senescence is reduced in PD, thus the enhanced proinflammatory cytokines may lead to the DA neuron apoptosis.

### 4.3. Microglial

Microglia are resident macrophage-like autoimmune cells in the central nervous system. It accounts for 10% of the total glial cells in the brain of healthy people [75, 117]. Especially in the SN, microglia content is the highest, about 4.5 times higher than in other parts. Under the trigger of pathology, microglia migrate to the apoptosis site and play a double-edged sword role, reduce or aggravate the injury. Microglia performed these tasks in two primary states: the resting and activated states, which can be distinguished from each other. In a resting state, microglia continue to wander in the surrounding environment and perceive pathology. Once the nervous system is attacked and pathological changes occur, microglia will change to an activated state [118]. In the brain of cadaveric patients diagnosed with PD dementia, HLA-DR-positive microglia increased significantly in the hippocampus, accompanied by decreased choline acetyltransferase activity in the cortex [119].

#### 4.3.1. Receptor-Mediated Apoptosis Related to Microglia

During the progression of PD, the activation of microglia can promote the inflammatory reaction and lead to the loss of DA neurons. For example, reactive oxygen species (ROS) induce reduced nicotinamide adenine dinucleotide phosphate oxidase 2 (NOX2) and then promote the production of hydrogen peroxide (H2O2), which ultimately shows toxicity and induces irreversible DA neuron apoptosis [120].

Microglia can also present antigen-derived peptides to CD4+ T cells via MHCII to enhance inflammatory response and further promote the degeneration of DA neurons [121]. Tumour necrosis factor (TNF) and complement Clq produced and released by microglia can directly induce astrocytes to differentiate into A1 phenotype and lead to DA neuron apoptosis [122, 123]. Activated microglia can also cause large-scale oxidative stress in DA neurons by producing nitric oxide (NO) and superoxide.

In addition, there is mutual induction between *α*-synuclein and microglia. Phagocytosis of *α*-synuclein oligomer mediated by FC7 receptor can induce microglia to transform into proinflammatory phenotype and then release a variety of cytokines, such as interleukin-1 (IL-1), interleukin-6 (IL-6) and TNF-*α*, and cyclooxygenase-2 nitric oxide synthase, free radicals. Moreover, compared with the monomer form of *α*-synuclein, the reaction caused by polymer *α*-synuclein is stronger [124].

#### 4.3.2. *α*-Synuclein Accumulation Related to Microglia

Study also found that microglia activated by *α*-synuclein can aggravate the loss of DA neurons in vitro, suggesting protein can play a role in neurodegeneration by activating microglia. C-terminal-truncated *α*-synuclein is the most potent inducer of neurotoxic behavior of microglia [125]. The specific conformation and specific mutation of extracellular *α*-synuclein can directly activate microglia. In BV2 microglia, *α*-synuclein fibrils and early-onset PD-related mutations induce a more robust immune response [126]. The activation of microglia is also involved in the pathological progression of *α*-synuclein. In pathological process, the cell activation and inflammation persist, and the clearance of microglia to the pathological form of *α*-synuclein is slowed. The mechanism may be that the misfolding and aggregation of *α*-synuclein can apoptosis the intracellular lysosome system, resulting in the gradual accumulation of undegraded *α*-synuclein, and finally lead to the physiological dysfunction of microglia and *α*-synuclein can also affect the permeability and function of other organelles by combining with lipids. It caused a decrease in phagocytosis of microglia [127]. In summary, the progress of PD may be driven by a vicious circle between dead neurons and microglia caused by oxidative stress, mitochondrial autophagy, and autophagy dysfunction, *α*-synuclein accumulation and proinflammatory cytokine release [128].

#### 4.3.3. Gene-Mediated DA Apoptosis Related to Microglia

Some studies have shown that LRRK2 knockout can reduce the oxidative stress and morphological changes of primary microglia induced by LPS [129, 130]. Manganese exposure can induce DA neuronal apoptosis and activate microglia. Inhibition of LRRK2 can effectively reduce the effect of manganese on microglia and restore the autophagy function [131]. Studies have shown that LRRK2/microglia increase migration behavior and change the response to fractalkine (CX3CL1) may mediate this phenotype [132]. Some studies have also found that LRRK2 affects mitochondrial function in microglia in a kinase-dependent manner through Drp1 and promotes the inflammatory response of microglia [133]. LRRK2 is involved in the internal regulation of microglial activation and lysosome degradation [134]. In G2019S knockout mice, iron deposition in microglia increased significantly after LPS injection into the striatum, accompanied by ferritin accumulation. Microglia derived from iPSC in patients with LRRK2 mutation G2019S transfer transferrin to lysosomes near the nucleus under proinflammatory conditions [135]. LRRK2 may regulate microglial cytoskeleton and vesicle transport under pathophysiological conditions [136]. Microglia from PD patients with LRRK2-G2019S mutation also increase phagocytosis through cytoskeleton remodelling factors [137] PD-related mutations in LRRK2 may affect the balance between microglia and *α*-synuclein, leading to cell dysfunction and neurodegeneration [134].

In addition, many other PD-related gene mutations may also affect the function of microglia. Plasma inflammatory markers and cytokines, including IL-8 and macrophage inflammatory protein-1 *α* [74], are increased in patients with GBA mutations in PD. The accumulation of GBA may also activate complement, which aggravates microglia-mediated neuronal dysfunction [138]. Indeed, one study observed that systemic GCase inhibition increased the accumulation of *α*-synuclein in the SN and upregulated complement C1q [139].

PINK1 deficiency can lead to loss of DA neurons and early apoptosis of mitochondrial function and morphology in zebrafish. The expression of zebrafish TigarB, human zebrafish homologue, tp53-induced glycolysis, and apoptosis regulator TIGAR was significantly increased in pink (- / -) larvae. Antisense TigarB inactivation leads to complete normalization of mitochondrial function, thereby saving DA neurons in pink (- / -) larvae. Pink (- / -) larvae also have prominent microglial activation, but the decrease of microglia cannot save the loss of DA neurons. It is considered that the activation of microglia is the critical factor in the pathogenesis of the disease [140]. In addition, parkin may play an essential role in microglia by regulating ubiquitin. The absence of parkin exacerbates inflammation and promotes the survival of activated microglia, leading to chronic neuroinflammation [141]. It has been found that parkin is involved in regulating mitochondrial autophagy, mitochondrial biogenesis, and mtDNA maintenance pathways, thus protecting midbrain neurons from neuroinflammation and degeneration [142] (Table 1).

### 4.4. NK Cell

NK cells are critical autoimmune cells in the body, and their origin is unclear. It is generally believed that they are derived from bone marrow and mature depending on the microenvironment of bone marrow [143]. They are widely found in lymphoid and nonlymphoid tissues, accounting for 10% of the total number of circulating cells 15% [144]. In recent years, many functions of natural killer cells have been discovered, such as reducing inflammation, forming immune memory, and regulating the function of antigen-presenting cells [145].

*α*-synuclein polymer was internalized and degraded by the endosome/lysosome pathway. In addition, NK cells can recognize and eliminate senescent cells, and the mechanism may be related to the interaction with senescent cells through granule exocrine secretion of granzyme, perforin, and production of INF-*γ* [146]. At present, it is still unknown whether NK cells can be cleared against *α*-synuclein-loaded cells. In addition, NK cells also have the effect of relieving neuroinflammation. Studies have shown that NK cells can decrease MHCI molecules' expression on activated microglia through interaction with microglia, produce cytotoxicity to activated microglia, and reduce the production of proinflammatory factors to alleviate neuroinflammation [147]. Microglia are continuously activated under *α*-synuclein loading [148]. And NK cells may also reduce microglial activation by scavenging *α*-synuclein. Li et al. found that the presence of MiR207 in the exocrine body of NK cells can reduce the release of proinflammatory factors from astrocytes and alleviate the symptoms of stress in mice [149].

The decrease of NK cells in autoimmune encephalomyelitis leads to the increase of autoreactive T cells and the enhancement of inflammation-related diseases [150]. NK cells can improve the symptoms of autoimmune diseases by releasing IFN-*γ* and play a neuroprotective role [151, 152]. The corresponding gene microarray analysis of IFN-*γ* shows that IFN-*γ* can promote the expression of genes related to protein degradation (ubiquitin D) and proteasome degradation (proteasome subunit *β* 9), suggesting that IFN-*γ* may promote lysosome digestion of excessive *α*-synuclein [153]. The decrease of IFN-*γ* produced by NK cells in the elderly population may be one of the reasons for the high incidence of PD in the elderly [154].

## 5. Autoimmune Cells in the Gut-Brain Axis of DA Neuron Injury in PD

### 5.1. B Cells and Plasma Cells

In peripheral system, the effect of intestinal flora on the number and function of B cells has been reported for a long time. For example, the structure of intestinal flora can regulate the differentiation, maturation, and activation of B cells. Dietary changes and probiotic therapy have been shown to control the number and function of B cells [155], while altering the intestinal microenvironment. In the central nervous system, many studies have also suggested that changes in intestinal colonies also seem to affect the state of central B cells profoundly.

First of all, it was found that intestinal IgA plasma cells were observed in the parenchyma of the central nervous system in mice with multiple experimental sclerosis. Studies based on neuroimaging also suggest that peripheral developing B cells may enter the dura mater from the skull's bone marrow [156–158]. A large number of plasma cells [159], CD8T cells, CD4T cells, NK cells, and Foxp3+ regulatory T cells [160] were colonized in the dural venous sinus wall [161]. After the intestinal barrier was destroyed, the number of IgA+ plasma cells and B cells around the paranasal sinuses increased. In addition, a subgroup of plasma cells in the sinus wall of the dural vein can coexpress the junction (J) chain and secrete polymerized IgA, similar to that secreted by intestinal plasma cells. And this phenomenon almost does not exist in mice that eradicate intestinal flora and can realize lgA resecretion of plasma cells in the dural venous sinus wall through the recovery of intestinal flora [161]. Moreover, central autoimmune cells, including B cells and T cells, can promote neuroinflammation by activating microglia and producing proinflammatory cytokines and oxidative stress products [162]. In the specific pathway mechanism, it is found that Phlorizin (PZ) not only improves the structure and diversity of intestinal microorganisms but also regulating the interleukin-1*β*/inhibitor of nuclear factor-kappa B alpha/nuclear factor-kappa-light-chain-enhancer of activated B cells signalling pathways in brain tissues, thus playing a role in the regulation of neuroinflammation. In the end, it may regulate DA neuron apoptosis and PD progression, indicating that intestinal microorganisms may regulate DA neuron apoptosis and PD progression by affecting the immune function of central B cells [163].

### 5.2. T Cells

In addition to the most apparent motor syndrome, most PD patients perform various nonmotor clinical manifestations. Among nonmotor images, gastrointestinal dysfunctions are the most common, which could be regarded as necessary as potential early biomarkers of PD since they are ubiquitously and typically found among patients at earlier stages.

**Figure 3:**
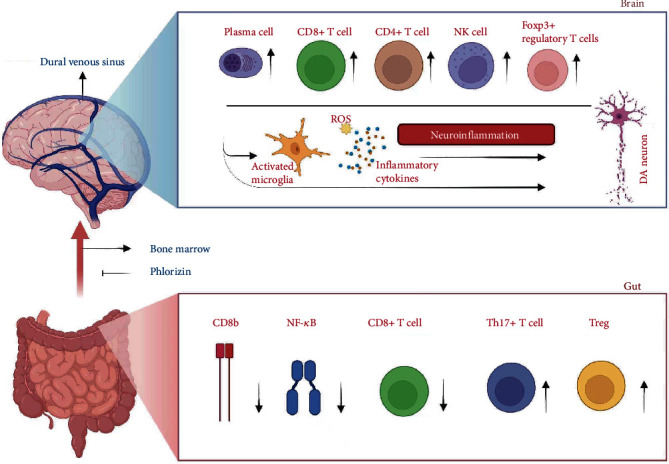
Autoimmune cells in the gut-brain axis of DA neuron injury in PD. The variation of autoimmune cell composition in gut mucosal, which travels through bone marrow into the dural venous sinus, constitutes the primary source of antigens driving immune response (including the release of inflammatory cytokines) and DA neuron apoptosis.

Constipation and inflammation of the gut mucosa are the most aberrant gastrointestinal dysfunctions, with associated pathological features including the loss of neurons of the enteric nervous system and the generation of Lewy bodies in the gut. Research has demonstrated that secretion levels of inflammatory markers, including CD8 B and NF-*κ*B p65, were significantly higher in PD patients' colon biopsies compared with HCs, and decreased levels of DA markers associated with colitis were observed in CD8+ T cells depletion [164]. A significantly high level of Th17 and Treg cells infiltration in PD patients with constipation was observed compared with that in patients without constipation (*P* < 0.001). Among all PD patients with constipation, the frequency of Th17 and Treg cells in STC was the highest [165]. These indicate that T cell immune response triggered in PD colon mucosa is indispensable with DA neurodegeneration in PD (Figure 3).

## 6. Therapeutic Prospects

The researchers immunized the B cell epitopes of *α*-synuclein to induce high titers of antibodies that could bind to three B cell epitopes associated with pathological *α*-synuclein deposition. Finally, they found that the resulting antibodies could reduce *α*-synuclein deposition and neurodegeneration [166]. In addition, ten monoclonal antibodies against *α*-synuclein protein were extracted from memory B cells of patients with PD, some of which showed functional activity in synuclein inoculation test in vitro and recognized pathological Lewy bodies in tissues of patients with PD [167]. Finally, there was increased activity of MAO-B in B cells of patients with PD and could be sensitively detected by a U1 small molecular probe [168]. All these suggest that B cell pedigree may be a potential biomarker for diagnosis and treatment of PD.

As for T cells, T cell receptors (TCRs) of *α*-synuclein-specific T cells have been mapped in PD patients. Results showed that TCR repertoires were specific to each PD patient. The probable reason for it is the difference in HLA expression. Thus, antigen-specific TCRs may be considered a therapeutic target for PD patients [169]. CCR5, the properties of C--C chemokine ligand 5, has been previously reported to participate in the activation of microglia and in the infiltration of T cell and NK cells, which could either result in neuroinflammation or DA neuron apoptosis [170, 171]. Several studies have identified CCR5 inhibitors' good physicochemical and pharmacokinetic properties in treating T cell-induced DA neuron apoptosis. Maraviroc, a blocker of CCR5, protects the central nervous system from T cell infiltration and microglial activation [172]. HMGB1 A box, a competitive inhibitor protein of HMGB1, which has been proved to aggravate the inflammatory response in PD, inhibits the Th17 ratio in CD4+ T cells and T cells infiltration in MPP+-induced animal model via modulating CD200-CD200R signal pathway [173]. Besides Maraviroc, adipose tissue-derived mesenchymal stem cells (Ad-MSCs) also reduce CD4+ T cell infiltration and inhibit the CD4+ T cell differentiating to Th17. The expression of LIF, an anti-inflammation protein, was significantly increased after the usage of Ad-MSCs [174]. Researchers also proposed that FK506 (an immunosuppressant) capsuled into the outer layer of alginate beads could reduce T cell response [175]. Enhancing the ability of Treg is a potentially helpful method to alleviate DA neuron apoptosis. Granulocyte-macrophage colony-stimulating factor (GM-CSF) is an essential immune regulator that increases the number of regulatory T cells (Tregs) and plays a neuronal protective role in PD patients [176]. A shift in CD4(+) T cell gene expression towards an anti-inflammatory phenotype corresponded with decreased microgliosis and increased DA neuronal cell survival. mPDm608 protected MPP+-treated mouse primary mesencephalic neurons in vitro by shifting CD4(+) T cell gene expression towards an anti-inflammatory phenotype and improving DA neuronal cell survival, mPDM608 elicited a neuroprotective peripheral immune transformation [177]. Additionally, LBT-3627, a vasoactive intestinal peptide receptor-2 (VIPR2) peptide agonist, improves the activity of Tregs instead of number [178]. However, the number or activity of Tregs improved alleviates the DA neuron apoptosis. Direct neuroprotective effects of improving autophagy in PD were also previously reported. Celastrol and dendric cell (DC) vaccine adjuvants induced autophagy, leading to a Th2-specific immune response and recruiting peripheral leukocytes to the brain [70, 179]. These results suggest that T cell lineage may be a potential biomarker for PD diagnosis and treatment.

Chlorogenic acid, a type of phenolic acid, has been demonstrated to have an antiapoptotic, anti-inflammatory, antioxidative, and neuroprotective properties [180–182]. Recently, some studies proposed CGA may alleviate the impairment of motor and inhibit the development of PD via inhibiting the activation of proapoptotic proteins including Bax and caspase-3, while elevating the expression of antiapoptotic protein like Bcl-2 [182]. Additionally, immune cells have been validated in CGA-mediated neuroprotective process, by improving the secretion of IL-10, an anti-inflammatory cytokines and inhibiting tumour necrosis factor-*α* and interleukin (IL)-1*β* [180].

It has been reported that microglia-mediated neuroinflammation may be an essential factor in the occurrence and development of PD. There is a relatively high inflammatory expression in brain tissue, cerebrospinal fluid, and blood of patients with PD [183]. Therefore, targeting activated microglia may be a potential target for treating PD [184]. In addition, M2 activation of microglia has a neuroprotective effect, and we can inhibit neuroinflammation by increasing the M2 polarization of microglia. For example, vitamin D can regulate the transition of microglia to M2 and play a neuroprotective role [185]. It has been reported that fingolimod (2mg/kg) can reduce the activation of microglia by BV-2 microglia treated with MPTP and 1-methyl-4-phenylpyridine (MPP). *Mucuna pruriens* was also found to have neuroprotective effect and immunosuppressive properties [186], and it possess a variety of pharmacological properties including antioxidant and anti-Parkinsonism effects; studies have shown that *Mucuna pruriens* significantly inhibited the release of inflammatory mediators including nitric oxide (NO), IL-1*β*, IL-6, and TNF-*α* in LPS-induced BV2 microglial cells [187]. However, whether it regulates microglia and DA neuron apoptotic by affecting oxidative stress is unclear. But overall, *Mucuna pruriens* can protects the DA neurons from the NO injury in substantia nigra [188]. In addition, in MPP+-treated BV-2 cells and primary microglia, fingolimod significantly decreased the phosphorylation level of PI3/K/Akt/GSK3 *β* signalling pathway, ROS production, and p65 phosphorylation by inhibiting NLRP3 inflammatory bodies, suggesting that fingolimod may be a strategy against PD [189]. The kinase activity of mutant LRRK2 also increases mitochondrial fission in microglia, resulting in impaired mitochondrial dynamics and higher production of TNF-*α*, which can be saved by LRRK2 kinase inhibitors [133]. It is suggested that LRRK2 kinase inhibitors have the potential to reduce the occurrence of neuroinflammation and play an anti-PD role. Finally, oxidative stress induced by microglia is an important factor in the deterioration of PD, *Withania somnifera* root extract have shown to counteract the prooxidants and their associated oxidative stress in the PD model [190], so it may play a role in the treatment of PD. Specific perspectives on T cell, B cell, and microglia-mediated PD treatment can be found in Table 4.

On the other hand, ursolic acid (UA), a natural pentacyclic triterpenoid compound, has shown protective activity in several experimental models of brain dysfunction by inhibiting oxidative stress, inflammatory responses, and inhibiting apoptotic signalling pathways. Yun et al. found that rotenone injection resulted in marked motor deficits and prodromal nonmotor symptoms, accompanied by marked loss of DA neurons and deposition of aggregates such as p62 and ubiquitin in the substantia nigra and striatum. Combined injection of UA can ameliorate all pathological changes caused by rotenone [191]; this suggests that UA may reduce neuronal apoptosis by regulating neuronal autophagy, thereby improving the symptoms and delaying the progression of Parkinson's disease. Peng et al. found that UA exhibited mitochondrial rescue effects in a Parkinson's model through activation of glucocorticoid receptors and increased Akt phosphorylation [192]. In addition, UA exhibited potent anti-inflammatory activity in an MPTP-induced Parkinson's disease model [193] and ameliorated behavioral deficits and protected DA neurons in MPTP-poisoned mice [188]. However, the potential of UA in the treatment of autoimmune cells remains to be explored.

On recent advancements, some mechanisms revealed may be key points for future PD therapeutic. The role of B cells in the progression of PD has been increasingly emphasized; B cells in the peripheral circulation are associated with PD progression and affected by the gut microbiot and may promote neuroinflammation and DA neuron apoptosis through the blood-brain barrier [16]. Therefore, blocking the entry of B cells into the blood-brain barrier may be the key to future research and treatment; on the other hand, Th1, Th2, and Th17 cells drive the proinflammatory response, while Treg cells play an antioxidant and immunosuppressive role [20, 21]. In therapy, scavenging of mtROS in Tregs of mice reversed DNA damage response and prevented Treg cell death, while attenuating the Th1 and Th17 autoimmune responses [194]. These findings highlight an unrecognized role of mitochondrial oxidative stress in defining Treg cell fate during autoimmunity, which may facilitate the design of new immunotherapies for PD mediated by autoimmune cell. In particular, some clinical trials from the perspective of autoimmune cells are being registered in recent years, which will be expected to further promote the treatment progress of PD (Table 5).

## 7. Conclusions

**Figure 4:**
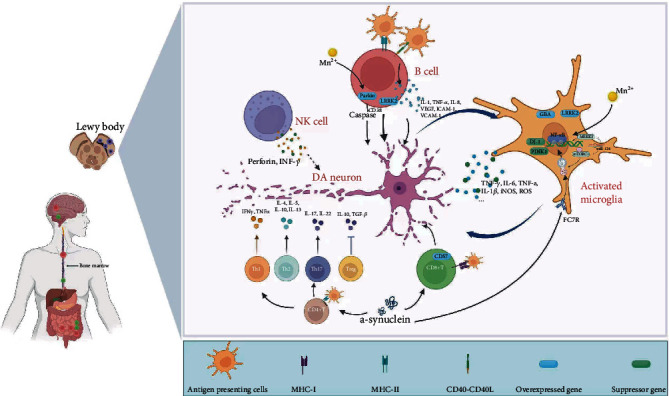
The mechanism of DA neuron injury is mediated by autoimmune cells. Autoimmune cells can enter the central nervous system through bone marrow from gut mucosal, located in the dura mater, midbrain, and other parts, and release various pathological factors to damage DA neurons through antigen presentation.

In conclusion, these studies indicate that B cells, T cells, NK cells, and microglia are the most important autoimmune immune cells responsible for the DA neuron apoptosis in PD and are widely involved in the core mechanism of PD initiation (Figure 4). The signalling pathways and molecular factors involved in autoimmune cells to DA neuron apoptosis have become an important research method to identify PD's pathogenesis. Research on autoimmune cells is expected to be an essential means to alleviate the progression of PD. For example, CCR5 inhibitors have good physicochemical and pharmacokinetic properties in treating T cell-induced DA neuron apoptosis; vitamin D can prevent microglia activation, thereby preventing DA apoptosis and playing a neuroprotective role.

Studies have shown that the regulation of signalling pathways and molecular factors involved in the pathological progression of autoimmune cells can effectively improve the DA neuron apoptosis caused by it. This paradigm is moving from theory to reality as a potential target for developing new drugs to treat PD. Focusing on these signalling pathways and molecular factors involved in the autoimmune response will help to understand the occurrence and development of PD better. Ongoing research in this area may open a new door for developing pharmacological strategies to prevent and alter the pathogenesis of PD.

## Figures and Tables

**Table 1:** The immune landscape of T cells in Parkinson's disease.

Species of autoimmune cell	Status of autoimmune cells in PD	Sample source	Pilot study	Reference
T cells	↓/CD4 T lymphocytes ↓/CD8+ T lymphocytes ↓/CD4/CD8 ratio	Serum	8 PD vs. 6 HC	[23]
	↑/Th1 ↓/Treg	Serum	20 PD vs. 20 HC	[24]
	↓/naïve CD4 T cells, *γδ*T cells, and iNKT cells ↑/NK cells	Serum	47 PD vs. 47 HC 205 PD vs. 233 HC	[25] [26]
	↑/cTfh ↓/cTfr	Serum	26 PD vs. 26 HC	[27]
	↑/Treg	Substantia nigra	205 PD vs. 233 HC	[26]
B cells	↓	Serum	8 PD vs. 6 HC	[12]

**Table 2:** The gene mutation of autoimmune cells associated with DA neuron apoptosis in PD.

Species of cells	Gene mutation	Changes in gene expression in PD	The immune response of autoimmune cells to PD “triggers”	Effect to DA neuron apoptosis	Reference
T cells	JKAP	↓	↓/Th1 and Th17 cell proportions	Alleviate	[67]
	PINK1	↓	↑/infiltration of CD8+ T cells	Aggravate	[51]
	ICAM1	↑	↑/infiltration of CD4+/CD8+ T cells	Aggravate	[68]
	LFA1	↑			
	GZMA, GZMB, GZMH, NKG7, KLRC3, and KIR2DL3	↑		Aggravate	[23]
	LRRK2	↑	↑/microglial activation ↑/CD4 and CD8 T cell recruitment	Aggravate	[69]
	Rab	↓	↓/*α*-synuclein aggregation ↓/*α*-synuclein-specific T cell responses	Alleviate	[70, 71]
B cells	LRRK2	↑		Aggravate	[58]
	Parkin	↑		Aggravate	[60, 61]
	RAG2	↓		Alleviate	[65, 66]
Microglia	LRRK2	↑	↑/TNF-*α*, IL-6, and NO	Aggravate	[72, 73]
	GBA	↑	↑/IL-8, MIP-1*α*	Aggravate	[74]
	Parkin and pink1	↓	↓/IL-10, ↑/IL-1*β*, IL-18, and ROS	Aggravate	[75]
	PARK7	↓	↑/IL-1*β* and IL-6	Aggravate	[76]

**Table 3:** Receptor-induced DA damage related to autoimmune cells in PD.

Species of cells	Receptor	The immune response of autoimmune cells to PD “triggers”	Effect on DA neuron apoptosis	Reference
T cells	DJ-1	↓/IFN-*γ*,I-TAC	Alleviate	[82]
	DRD2	↓/shift of CD4+ T cells to Th1 and Th17 cells	Alleviate	[83]
	TCRb	↓/MHCII response to *α*-synuclein	Alleviate	[84]
	CD4			
	CTLA-4	↓/IL-10 and IL-4	Alleviate	[84]
	PD-1			
	IL-17R	↑/IL-17, IL-22, TNF-*α*, IL-1*β*, IFN-*γ*, and iNOS	Aggravate	[85]
	TLR7	↑/recruitment of T cells	Aggravate	[86]
	TLR8			
	MHC-I	↑/infiltration of CD8+ T cells	Aggravate	[51]
	MHC II	↑/*α*-synuclein accumulation ↓/shift of CD4+ T cells to Th1 and Th17 cells	Aggravate	[65, 87, 88]
B cells	CD40	↑/TRAF, PI3K, IL-1, TNF-*α*, IL-8, IFN-*γ*, and iNOS	Aggravate	[12, 80]
Microglia	TREM2	↓/IL-1*β*, iNOS, IL-6, ↑/IL-10, and Arg-1	Alleviate	[76]
	GPR30	↓/TNF-*α*, IL-1*β*, 和IL-6	Alleviate	[89]
	MT1	↓/IL-6, TNF-*α*	Alleviate	[90]
	CD200R1	↑/microglia activation	Alleviate	[91]
	CR3	↓/INOS, TNF-*α*, IL-1*β*, and ↑/Arg-1,	Alleviate	[92]
	MHC II	↑/IL-2 and TNF-*α*	Aggravate	[93]
	TRL-4	↑/IL-1*β*, iNOS, IL-6, ↓/IL-10, and Arg-1	Aggravate	[94]
	DJ-1	↓/IL-1*β* and IL-6	Alleviate	[75]

**Table 4:** The therapeutic prospects of immune cells in PD.

Species of cells	Therapeutic method	Mechanism	Effects	Reference
B cells	Antibody from B cells	Bind to three B cell epitopes associated with pathological *α*-synuclein deposition	↓/*α*-synuclein deposition and neurodegeneration	[166, 167]
T cells	Maraviroc	Blocker of CCR5, alleviate T cell infiltration and microglial activation	↓/DA neuron apoptosis	[170–172]
	Ad-MSCs	Reduce CD4+ T cell infiltration; inhibit the CD4+ T cell differentiating to Th17	↑/the expression of anti-inflammation protein LIF	[174]
	FK506	Reduce T cell response	↓/DA neuron apoptosis	[175]
	GM-CSF	Increase the number of Tregs	↓/DA neuron apoptosis	[176]
	mPDm608	Promote the shift in CD4(+) T cell gene expression towards an anti-inflammatory phenotype	↓/DA neuron apoptosis	[177]
	LBT-3627	Improve the activity of Tregs	↓/DA neuron apoptosis	[178]
	Celastrol	Induce autophagy; lead to a Th2-specific immune response; and recruit peripheral leukocytes to the brain	↓/DA neuron apoptosis	[70]
	DC vaccine adjuvants	Induce autophagy; lead to a Th2-specific immune response; and recruit peripheral leukocytes to the brain	↓/DA neuron apoptosis	[179]
Microglia	Vitamin D	Regulate the transition of microglia to M2	↓/DA neuron apoptosis	[185]
	Fingolimod	Inhibit NLRP3 inflammatory bodies	↓/DA neuron apoptosis	[189]
	LRRK2 kinase inhibitors	Reduce impaired mitochondrial dynamics; Reduce the production of TNF-*α*	↓/DA neuron apoptosis	[133]

**Table 5:** Vaccines and drugs in clinical trials or approved for PD based on autoimmune cells.

Species of autoimmune cell	Drugs	Mechanism	Effect	Reference
T cells	Glutathione	Regulates T cell activation and propagation	↓/oxygen radical	NCT01398748 [195]
Microglia	Hypoesttoxide	Decrease activation of microglia and astrocytes	↓/inflammatory cytokines ↓/DA neuron apoptosis	NCT04858074 [196]
	WIN-1001X	Blocking RhoA/ROCK2 signalling pathway	↓/inflammatory cytokines ↓/DA neuron apoptosis	NCT04220762 [197]
	NLY01	Reducing activation of microglia, preventing healthy astrocytes from turning into destructive astrocytes	↓/DA neuron apoptosis	NCT04154072 [198]
	Caffeine	Attenuated *α* -synuclein-induced microglial activation and astrocytosis in mice	↓/inflammatory cytokines ↓/DA neuron apoptosis	NCT01738178 [199]
